# Modifications of a Parenting Program in the Context of Scaling-Up and Scaling-Out: Documenting Furaha Teens in Tanzania Using FRAME

**DOI:** 10.1007/s11121-025-01852-5

**Published:** 2025-10-30

**Authors:** Yulia Shenderovich, Mackenzie Martin, Jamie M. Lachman, Samwel Mgunga, Esther Ndyetabura, Joyce Wamoyi

**Affiliations:** 1 Wolfson Centre for Young People’s Mental Health, Cardiff University, Cardiff, UK; 2 Centre for Development, Evaluation, Complexity and Implementation in Public Health Improvement (DECIPHer), School of Social Sciences, Cardiff University, Cardiff, UK; 3 Community-University Partnership for the Study of Children, Youth and Families, School of Public Health, University of Alberta, Edmonton, Canada; 4 Department of Psychiatry and Behavioural Neurosciences and the Offord Centre for Child Studies, Faculty of Health Sciences, McMaster University, Hamilton, Canada; 5 Department of Social Policy and Intervention, University of Oxford, Oxford, UK; 6 MRC Social and Public Health Sciences Unit, University of Glasgow, Glasgow, UK; 7 Parenting for Lifelong Health, Oxford, UK; 8 Centre for Social Science Research, University of Cape Town, Cape Town, South Africa; 9 Department of Sexual and Reproductive Health, National Institute for Medical Research Mwanza Research Centre, Mwanza, United Republic of Tanzania; 10 Pact Tanzania, Dar es Salaam, United Republic of Tanzania; 11 National Institute for Medical Research, Mwanza, United Republic of Tanzania

**Keywords:** Program adaptation, Dissemination, Scale-up, Implementation science, Low-resource setting, Low- and middle-income countries, Parenting program, Adolescent health

## Abstract

Program adaptations or modifications are often necessary to suit local contexts, populations, and resources available. Despite the frequency with which program modifications are made in practice, they are rarely systematically recorded and reported comprehensively, particularly in the context of scale-up delivery led by implementers and in low- and middle-income countries. We use the FRAME framework to document the modifications of a parenting program called Parenting for Lifelong Health for Parents and Adolescents, locally known as Furaha Teens, which was delivered to over 30,000 families in Tanzania in 2020–2021. We draw on thematic analysis of 12 focus groups and 67 semi-structured interviews with program facilitators, coaches, coordinators, and managers (164 participants). Both proactive and reactive modifications were made to the program context and content. Proactive modifications included delivering the program as part of a wider package of services for families with adolescent girls, focused on HIV prevention, and adding HIV-related content. Both proactive and reactive modifications were made to make the material more acceptable to participants, such as by translating into local languages. Modifications to condense the number and frequency of sessions were reactively made by implementers to meet delivery timelines, particularly due to COVID-related closures. Study findings suggest that a range of program modifications may be required to scale programs to large cohorts as well as new contexts. To ensure successful delivery at scale, funders can support implementers in learning from the modifications and encouraging reflection on whether and how modifications affect program fidelity.

**Supplementary Information:**

The online version contains supplementary material available at 10.1007/s11121-025-01852-5.

## Introduction

A recent scoping review of 145 studies on parenting programs delivered in community settings found that 40% of the studies reported program adaptation (Pinto et al., 2023). While program adaptation is often necessary to fit new contexts, populations, and resources available (Escoffery et al., 2019), adaptations are still rarely recorded and reported comprehensively. This is especially the case in implementation outside of the context of structured research studies, such as program delivery in routine service delivery settings (Chambers & Norton, 2016), where drift from the original design may occur over time (Aarons et al., 2012a, 2012b). Therefore, researchers have called for systematically tracking adaptations (Baumann & Cabassa, 2020; Shenderovich et al., 2021), particularly in the context of scale-up (Power et al., 2019). Scale-up is when a program is delivered to more participants (horizontal scaling) and/or embedded in longer-term delivery systems (vertical scaling) (WHO, 2010).

Systematic reviews of adaptations of public health interventions and adaptation guidance (Escoffery et al., 2019; Movsisyan et al., 2019), interviews with researchers, funders, and implementers on the topic of adaptation (Copeland et al., 2021) have all highlighted varied definitions of adaptation. We draw on the Framework for Reporting Adaptations and Modifications-Expanded, or FRAME (Stirman et al., 2013, 2019). FRAME uses “modification” as a global term for alterations to intended program delivery that are either proactive (i.e., planned, also called adaptations) or reactive (i.e., responsive to unanticipated circumstances). For each modification, FRAME recommends recording whether it was proactive or reactive, the level at which the modification was made, its timing, reasons, and who was involved in the decision-making.

While the literature has focused on proactive modifications, there is increasing recognition that reactive modifications are common during the implementation and scaling of interventions (Moore et al., 2013). Reactive modifications are often needed and valuable, for instance, to ensure intervention acceptability and engagement in new populations (Copeland et al., 2021). Modifications can also improve the feasibility of program delivery for practitioners, for example, a US study found a positive association between how much teachers felt they could modify the school-based health program and their reported program dosage delivered (Eisman et al., 2020). However, some reactive modifications may hinder intervention effectiveness.

FRAME can be used to document modifications in areas such as program context and content. For content modifications, FRAME advises assessing whether they align with fidelity to the original intervention. Greater fidelity is generally hypothesized to be associated with better participant outcomes. In a recent systematic review, some but not all studies of parenting programs focused on child behavior showed this association (Martin et al., 2023a, 2023b). Increasingly, fidelity is considered in terms of core functions and forms, where functions are the purposes of interventions, achieved via modifiable forms (activities) (Fox et al., 2025).

Reactive modifications are generally assumed to be less fidelity-consistent, although this has not been tested extensively and few studies have assessed the fidelity-consistency of modifications. A family program in the US used practitioner surveys for tracking adaptations, finding that 61% were not fidelity-consistent (Cooper et al., 2016). A study in Sweden coded focus group and interview responses of parenting program facilitators to identify modifications, which were rated on fidelity-consistency, based on whether a modification could help achieve any intervention functions (Pettersson et al., 2024). Most of the reported modifications, including reactive modifications, were fidelity-consistent, but fidelity-consistency was less frequent for modifications made without a clearly articulated reason.

In addition to fidelity-consistency, content modifications are classified in FRAME by their nature into categories, such as condensing, repeating, adding or removing program elements, and tailoring (meaning tweaking, refining). Finally, FRAME classifies the timing of modifications into four stages: pilot, implementation, scale-up, and sustainment. Our project was located within the scale-up stage, focusing on dissemination to many families. In addition, we explore program modifications in the context of being delivered in a new location (scale-out) (Aarons et al., 2017). FRAME was designed and originally used with psychosocial interventions in US healthcare, later expanding to new settings and regions, including prevention programs for children, within primary studies and literature reviews, including examining modifications due to the COVID pandemic (Fang et al., 2024).

### Background for the Current Study

The current study provides an example of horizontal scaling by focusing on the large-scale implementation of Parenting for Lifelong Health (PLH) for Parents and Adolescents, a group-based parenting program based on social learning theory and behavior change principles to reduce the risk of violence against adolescents. The 14-session program was developed and tested in South Africa with adolescents aged 10–18 in two pilot studies and a randomized trial (Cluver et al., 2016a, 2016b, 2018). The program includes weekly group sessions for adolescents and their parents/caregivers of approximately 3 h each, including discussions, role plays, experiential activities, and home practice. Sessions focus on topics such as spending time together, problem-solving, and resolving conflicts. Home visits are provided to participants who miss a session or require additional support. Trained community-based facilitators deliver the program to families. Trained coaches supervise facilitators, providing feedback to facilitators on their adherence to program content and competence in core facilitation skills (Martin et al., 2023a, 2023b).

The randomized trial (RCT) of the program in South Africa found reduced rates of violence against adolescents, improved positive and involved parenting, and improved supervision of adolescents (Cluver et al., 2018). In the trial, there were 552 participants, and on average 14 families per PLH group in the intervention arm (Shenderovich et al., 2018; Shenderovich et al., 2019). Following this trial, the program was disseminated in new settings, including in Tanzania where it is locally known as the Furaha Caring Families program for Parents and Teens (Furaha Teens). Implementation in 2017–2022 was led by Pact Tanzania as part of the Kizazi Kipya (or “New Generation”) Project, which was funded by USAID-PEPFAR Determined, Resilient, Empowered, AIDS-free, Mentored and Safe (DREAMS) initiative and aimed to reduce HIV infection incidence among adolescent girls and young women by providing family strengthening, HIV screening, sexual reproductive health training, and other services to caregivers and their adolescent girls aged 9–14.

In 2020–2021, Kizazi Kipya expanded Furaha Teens delivery with 444 community-based facilitators (schoolteachers and volunteers) and 70 coaches, to reach 75,061 participants (38,802 adolescents and 36,259 caregivers). Delivery took place in eight districts within three regions of Tanzania (Mbeya, Shinyanga, and Kagera). Clowns Without Borders South Africa (CWBSA)—a non-governmental capacity-building organization that supports those delivering the program in Africa—provided training to coaches and facilitators. Delivery was coordinated by Pact and managed by six local implementing partners (LIPs), local non-governmental organizations. There were three rounds of program delivery, and delivery was interrupted for several months due to the COVID-19 pandemic and associated school closures. This paper is part of a larger mixed methods study exploring the delivery of Furaha Teens within this scale-up (Martin et al., 2021). Results from the pre-post adolescent and caregiver outcomes are reported by Lachman et al. (2024). As part of the larger study, observational assessments of program fidelity found high rates of facilitator competence and adherence (Martin et al., 2025). Our research question in this paper is: “What are the modifications made as part of Furaha Teens scale-up in Tanzania, compared to the program evaluated in the RCT in South Africa, as reported in practitioner interviews and focus groups?”

## Methods

### Data Collection Procedures

We conducted semi-structured interviews and focus groups (overview in Table 1) with Furaha Teens implementers to explore pre-specified questions as well as emerging topics. Coaches and facilitators were approached by Pact and LIPs who asked for permission for their contact information to be shared with researchers. In each of the eight districts, the research team approached three facilitators and three coaches and asked for their help in recruiting colleagues for focus groups, with purposive sampling to ensure diversity. Recruitment was conducted to maximize participant variability based on (1) location, (2) timing of participation in program delivery, and (3) for facilitators, professional background, i.e., including both facilitators who were schoolteachers and volunteers. We expected that context may vary across locations and timing, thereby necessitating different intervention modifications, particularly due to COVID, while facilitator background may influence modifications because of previous training or ongoing work with the same communities in a different role (Shinde et al., 2020).

**Table 1:** Data collection overview

Category	Round 1		Round 2
	Interview (*N* = 36)	Focus groups (*N* = 12)	Interview (*N* = 31)
Coaches	10	4	10
Facilitators	10	8	12
LIP coordinators	5	-	4
CWBSA staff	2	-	1
Pact Tanzania staff	2	-	4

For potential participants who consented to be contacted, researchers provided detailed information about the study on the phone prior to seeking written informed consent. Following the focus groups, coaches and facilitators were invited to take part in a first round of interviews. Coaches and facilitators were selected for interviews based on their availability and interest. They included a mix of staff who were and were not vocal in the focus groups to include staff who might have had different levels of engagement in the program. Later, coaches and facilitators from the focus group who had not taken part in round one interviews were invited to a second round of interviews. Sampling for interviews from CWBSA and Pact Tanzania included all staff with relevant positions in the project. Program managers/coordinators from five LIPs were also invited to participate in the interviews. The first round of the qualitative data collection with coaches, facilitators, and coordinators included participants from five out of six LIPs, and the second round included participants from three out of six LIPs, due to feasibility considerations. We refer to the participants of this qualitative data collection collectively as implementers. Two experienced qualitative researchers conducted the interviews and focus groups. Our interview and focus group guides are available on OSF: https://osf.io/m5fu2/files.

The first round of qualitative data collection was used to identify program modifications, and the second round explored each modification in more depth. Focus group and interview questions on modifications were derived from the questions in FRAME, such as the timing of the modifications and their rationales. Focus groups lasted 90–120 min and interviews 60–90 min, with all focus groups taking place in person, and interviews both in person and on the phone or online. Participants received lunch as well as transportation to and from the meeting venues (value of approximately 5 USD). The interviews and focus groups took place during and after program delivery, so the responses included both concurrent and retrospective reporting of modifications.

### Analyses

Interviews and focus groups were conducted in Swahili. The audio recordings of the interviews and focus groups were transcribed, with transcripts labelled with participant IDs. Transcripts were translated into English and analyzed using NVIVO 12 qualitative analysis software. To identify program modifications, we reviewed intervention manuals and program delivery procedures as it was delivered in the randomized trial in South Africa, and in Furaha Teens in Tanzania. The coding framework was developed both inductively and deductively, based on the FRAME framework and the data (Braun & Clarke, 2021). At the start, double-coding was conducted using several transcripts, with coding differences discussed to agree shared approaches to further coding. Codes were created for each modification, and within that, sub-codes were focusing on the details of each modification (e.g., timing). Memos on different modifications captured researcher interactions with the transcripts and facilitated discussion. For content modifications, YS and JML (co-lead of the original intervention’s development) assessed fidelity-consistency, based on whether the modification supported one or more core program functions. Assessments were made independently by each rater and then discussed to reach consensus judgement.

### Reflexivity

The study team included individuals with experience in researching social programs and qualitative research in Tanzania and elsewhere. Several authors have been involved in the previous implementation and evaluation of PLH. Familiarity with the program helped identify modifications but may have also contributed to a more positive view of the program and its implementation, or a bias against modifications in favor of the original program version. We addressed this by including researchers who were not involved in the program development and discussing our findings inside and outside the team. The researchers and implementers worked closely to examine the program implementation, which may have helped discover modifications as rapport was gradually built.

## Findings

We present the findings using the questions outlined in FRAME. The illustrative quotes are from interviews as these provided a greater level of detail. See supplementary material for a summary of all the modifications identified (Table  1s).

### Context Modifications

#### Population

The original implementation was on community level with self-referrals as well as referrals from community members, social services, and schools (Cluver et al., 2018). As a proactive modification by Pact, the implementation of Furaha Teens was embedded within a larger HIV-prevention program, Kizazi Kipya, which included a package of health and social services. Implementers said the delivery of Furaha Teens as part of Kizazi Kipya may have influenced participation as some families were motivated by other components of Kizazi Kipya rather than Furaha Teens specifically:

there was that notion where a child knows that if she doesn’t attend these teachings [parenting program], we won’t be given a bag and uniform [other services in Kizazi Kipya]. (LIP 01)

[being part of a bigger package] also contributes to them getting a holistic service, where maybe the parenting aspect may have been [previously] missing […] In terms of the negatives […] to what extent are we respecting the right for people to participate or to choose to not participate, that is always the question that I ask our partners when they layer services like this. (CWBSA 04)

Second, the context of delivery with the PEPFAR-DREAMS initiative limited engagement to adolescent girls and their caregivers, whereas the intervention was designed for adolescents of any gender. Some participants and community members raised questions and concerns about the exclusion of boys. One facilitator reported that they made a reactive modification by also delivering the same intervention content to boys in separate meetings.

#### Format

##### Group composition

The program was designed to be delivered to one adolescent and caregiver per family. Some facilitators made reactive modifications during delivery by allowing or encouraging other adolescents or caregivers from the same family to participate. Facilitators explained that this modification was intended to increase family engagement with the intervention and avoid turning away other family members who came to sessions:

Since it’s the same family, sometimes if the mother isn’t attending, she will request the father to come and listen to what is been taught […] after sitting with my colleague we saw that it wasn’t good to tell another parent to go back home. (Facilitator 08)

In other cases, facilitators accommodated additional participants by inviting them to join as observers rather than active participants:

The father who was attending opted to come with mother so that she can now listen to what is being taught, so we said that those extra people that will be attending are allowed, but […] we didn’t involve them in our circle. (Facilitator 07)

##### Group size

With guidance from CWBSA, the group size was expanded to 20 families per group as a proactive modification. This meant that any given session could have 40 participants. Later on, reacting to the pandemic to mitigate risks related to transmission of COVID, some LIPs and facilitators reduced group sizes (e.g., by splitting the groups in two to about 10 families per group).

##### Format of home visits

In most cases, the implementers reported that the home visits proceeded without modifications. However, some facilitators noted combining multiple caregivers who missed sessions into a small group home visit catch-up:

And what they were doing in most cases, they were grouping together the families that were not present [at the previous session]. (Pact 03)

#### Personnel

The program manual does not specify the professional background of facilitators as it was explicitly designed for a range of facilitators, including lay community members. The randomized trial of the program in South Africa used a combination of lay workers and social workers. In the delivery of Furaha Teens, it was proactively decided by Pact that teachers and community workers would deliver the program for long-term sustainability:

government was positive and they cooperated but also we went further, even having facilitators who are teachers, government employees, you know supporting delivery of the intervention. (Pact 03)

Utilizing both types of facilitators brought advantages for program delivery. Participants commented that community facilitators were drawing on existing relationships in the community, and teachers were drawing on existing relationships with the adolescents and caregivers in their schools. However, there were also disadvantages, as teachers needed to balance program delivery with other work commitments. Over time, the initial allocation of staff was adjusted by the LIPs so that the teachers who acted as facilitators delivered the program either at their school or locations close to their school or residence. Delivery by teachers also presented some challenges in respect to existing teacher disciplinary practices:

Since the program believes in positive ways of parenting, you get that same teacher is the one punishing a child or beating a child at school during studies and it’s the same teacher who is later supposed to come and teach on minimal level of punishment to children, so you get there is conflict of interest in the information been delivered. (CWBSA 03)

### Content Modifications

#### Adherence to the Manual

Overall, implementers highlighted the importance of adhering to the intervention manual during program delivery, suggesting few reactive content modifications were made:

No change was done in the guideline [intervention manual]; we were not able because I think it’s like a curriculum that is guided so it’s a must [that] you carry out what is supposed to be done since they refer to it as evidence-based. (LIP 02)

Nonetheless, several proactive and reactive content modifications were made.

#### Inclusion of HIV Prevention Content

Prior to program implementation, reflecting the context of delivery within the PEPFAR-DREAMS initiative, the Furaha Teens content was modified by CWBSA in consultation with USAID to align with PEPFAR requirements by increasing its focus on HIV risk and protective behaviors. Modifications included (a) targeted discussion questions on sexual violence, HIV risk, and sexual and reproductive health added to role plays and activities; (b) additional activities aimed at the prevention of sexual violence and HIV risk; (c) additional training for facilitators on HIV risk prevention; (d) increased content on gender equality; (e) guidelines on how to respond to reported cases of child maltreatment; and (f) additional discussion on intimate relationships and sexual and reproductive health. We consider this set of modifications to be fidelity-consistent, as one of the aims of the program is to reduce adolescent exposure to risk in the community.

Facilitators also introduced their own HIV-prevention content during program delivery, including demonstrations on the use of condoms in the sessions. This was intended to reduce adolescent exposure to risk; however, qualitative data from caregivers suggests this was seen as embarrassing by some caregivers, therefore reducing acceptability (findings from caregiver and adolescent qualitative data reported in Wamoyi et al., 2025). Therefore, we classify this as fidelity-inconsistent.

#### Cultural and Linguistic Modifications

As a proactive modification, Pact and CWBSA translated the Furaha Teens facilitator manual into Swahili and culturally adapted it by using local character names and references. In addition to these proactive modifications to the manual, during delivery, further reactive modifications were made by facilitators to accommodate participants who did not speak Swahili. For example, in some regions, facilitators provided real-time interpretation for participants who spoke Sukuma, either by speaking participants’ preferred language or engaging other program participants to translate.

a few people don’t know Swahili […] we would continue going over those points that were hard in Swahili […] So you would find, maybe the neighbor understood the lesson, [then] they would discuss with each other, which makes it easier, so no one is left behind. (Coach 02)

Facilitators and participants also reactively introduced local songs for energizer activities in the sessions, which were seen as more engaging and acceptable, in place of the ones listed in the intervention manual. These proactive and reactive modifications can be classified as tailoring, and we consider them fidelity-consistent as they promoted family engagement with the program, and active engagement is consistent with program functions, given the program’s focus on the active participation of adolescents and caregivers and practicing new skills. These modifications are also consistent with facilitator skills required by the program, such as ensuring participants are comfortable and engaged in the session.

#### Frequency and Length of Sessions

Reactive modifications to the frequency and length of sessions were made by LIPs to complete program delivery within 1 or 2 months instead of 3 months. This included delivering the sessions two or three times a week instead of once per week. In many cases, this was related to delays because of school closures due to the COVID-19 pandemic:

… to follow the demands and requirements of the donor, we interfere with facilitators’ schedules and beneficiaries, requesting them to come up with a plan of completing by the second month. (Coach 07)

Session frequency was also increased reactively by facilitators in some groups to accommodate participant preferences, employment, and other livelihood-related activities. Decisions to change the frequency and timing of sessions were largely made by facilitators in consultation with community leaders and caregivers:

That suggestion [of two sessions a week] was from participants themselves, both parents and children – they are the ones who suggested [it], those are our customers […] so it’s a must you listen to what they want, and you implement it. (Facilitator 04)

These modifications are considered fidelity-inconsistent since the program was designed to allow sufficient time for caregivers and adolescents to apply skills learned during sessions. A key program component included a facilitated discussion in which participants share challenges experienced when applying new skills and are guided to identify potential solutions. Reducing the time in between sessions may have resulted in cognitive overload, in which participants were exposed to too many new concepts without sufficient time to integrate them into daily behaviors or reflect on their usefulness.

Following the pandemic closures, some LIPs also included a refresher on the sessions covered before the lockdowns, increasing the length of program sessions.

An example is you might find some classes usually arriving at four, but we ask them to come at three in order to achieve our goals. […] Then we would do a recap, [and] when we are done, we would move ahead with lessons. (Coach 02)

We consider the addition of refreshers as fidelity-consistent, as it allows more opportunities to reinforce the content.

#### Condensing Content

Some facilitators reported reducing the number of sessions by combining two or three sessions to meet the funder’s timelines (i.e., three program delivery rounds needed to be completed within one calendar year), especially to catch up after the pandemic-related delays. Some of the coordinators and coaches said they felt that combining sessions was fidelity-inconsistent, as the manual specified these as separate sessions and discouraged combining:

at the beginning it was realized some of them [teachers] had started the habit of merging lessons. (Coach 07)

Implementers reported that they saw delivering sessions more frequently as more fidelity-consistent than combining sessions because it allowed delivering the original number of sessions, albeit with closer spacing:

We cancelled issues with reducing lessons and so forth because, as for SOP [standard operating procedure], it states that no matter how urgent, it’s better to carry out today one lesson on Monday, another lesson on Friday. (Pact 02)

Particularly after COVID-related delays, Pact agreed with LIPs to combine some sessions.

Some facilitators also reported delivering home visits less often than specified in the manual (i.e., home visits were not conducted after every missed session), combining home visits for multiple sessions into one to reduce facilitator workload and discourage missing sessions. We consider the merging of sessions and home visits as fidelity-inconsistent because it likely decreased the overall content delivered and practice time offered.

## Discussion

Our study documented proactive and reactive modifications in an implementer-led parenting program delivered at scale in Tanzania. We captured modifications through extensive interviews and focus groups with implementers and found that implementers made proactive and reactive modifications to the program context and content. Our findings advance intervention adaptation research by illustrating how and why a program might be modified in practitioner-led large-scale delivery in a low-resource community setting.

Similar to previous research on parenting programs (Jürgensen et al., 2025; Pettersson et al., 2022), we found several content modifications aiming at linguistic and cultural tailoring made by implementers to increase program acceptability. We assessed these proactive and reactive modifications, such as facilitators’ use of local languages and songs, as fidelity-consistent. Such modifications, responsive to caregivers’ needs, may improve family engagement and, therefore, program outcomes (Lansford et al., 2022). Several context modifications, such as involving other family members in the sessions, were also targeting participant engagement and acceptability.

Furaha Teens was modified proactively based on its delivery as part of a package of services to families with adolescent girls. (Pettersson et al., 2022). Meeting the expectations on program focus and timeline associated with funding—a key factor for program sustainment (Birken et al., 2020)—was the reason reported for several proactive and reactive content and context modifications. Funding requirements motivated both the content modifications that we judged as fidelity-consistent, such as proactively adding HIV-related content, and fidelity-inconsistent, such as reactively combining sessions and reducing program duration, due also to the timelines aggravated by the COVID-19 pandemic. The modifications made in relation to COVID (e.g., group size, meeting frequency) share similarities with COVID-related changes reported by other parenting programs (Fang et al., 2024; Shenderovich et al., 2022).

A common reactive modification was shortening the program—either by combining the sessions or delivering them more frequently—so that the program designed to be delivered over 14 weekly sessions was sometimes delivered over 1 or 2 months. This modification was due to USAID-PEPFAR grant requirements to reach a targeted number of beneficiaries within a calendar year as well as movement restrictions and delays to implementation during the COVID-19 pandemic. Similarly, in a global review of adaptations of 42 public health evidence-based interventions (Escoffery et al., 2018), the authors found that more than half of the studies reported shortening the original program during routine delivery. Such modifications may be detrimental to fidelity and program effects by interfering with the intended behavior change process through reduced time to incorporate new skills or the omission of key activities (Cooper et al., 2016). Ensuring that programmatic targets can fit within funding timelines may reduce the likelihood of these modifications.

Our findings also provide context for the quantitative pre-post study of the adolescent and parent outcomes in Furaha Teens, which found improvements across multiple adolescent and caregiver outcomes, except for reduced positive parenting and parental support of education (Lachman et al., 2024). While the pre-post study lacked a comparison group, thus limiting interpretation of causal effects, it is possible that the modifications identified in the present study were either insufficient to adapt the program for the Tanzanian context or drifted too far from the originally tested program to sustain effects. Although there may have been other factors driving these potentially negative effects, such as the impact of COVID-19, these findings support the need for transparent reporting of program modifications to provide a more substantive understanding of why interventions may or may not produce similar results from more rigorously tested versions.

The modifications revealed by the qualitative data collection were not captured in the session observations, suggesting the importance of using multiple methods to study program implementation and identifying ways to assess both modifications and fidelity together (Basha et al., 2025). Modifications not being captured in fidelity assessments might explain inconsistent findings regarding the associations between facilitator fidelity and caregiver and adolescent outcomes reported in some studies of parenting programs, including with data from the present study (Martin et al., 2023a, 2023b, 2025). Modifications may not have been captured via our fidelity assessment as the fidelity measurement did not explicitly ask about modifications and covered only a small sub-sample of the sessions. As a result, the fidelity measurement does not reflect the modifications described herein.

### Implications for Prevention Science and Practice

There is growing recognition that program modifications are likely needed for scaling up and out. Preparing for modification is therefore necessary as part of planning for scaling up. Scale-up is likely to involve new delivery settings, personnel, participant groups, as well as new funding mechanisms, as was the case in our study. While in this project the PLH program was modified to contribute to the prevention of new HIV infections, in other contexts it has been modified to support other health outcomes, such as sexual reproductive health, caregiver and adolescent mental health, prevention of intimate partner violence, and child sexual abuse (Baerecke et al., 2024; Jocson et al., 2023). As well as proactive modifications, scale-up can include innovative reactive modifications by program facilitators, such as, in our study, accommodating the linguistic diversity of participants by engaging their peers, which can be integrated into standard operating practice.

Program funders can support adaptive management, so that reactive modifications can be accommodated, including innovations that can make the program work better with a new population or context (INSPIRE Working Group, 2021). Guidance documents and frameworks such as IDEA (Miller et al., 2020), MADI (Kirk et al., 2020), Dynamic Adaptation Process (Aarons et al., 2012a, 2012b), and ADAPT (Moore et al., 2020) can be used to guide decisions around modifications. There is also emerging evidence on how training focused on modifications may influence the modifications made by implementers (Zetterlund et al., 2025). Frameworks such as FRAME are a valuable tool for researchers and implementers to use when documenting program modifications. Funders need to be aware of the need for modification, support organizational learning to facilitate modifications, and provide time for implementers to pilot the modified intervention. Our work suggests that modifications are especially likely where a program is added to a package of services, like Furaha Teens was in the DREAMS package. Program duration is something that needs to be considered carefully at the start and, if possible, included with some margin for delays in the timeline. This may reduce the need for implementers to change the program due to timelines. Program developers can offer several program length options, from which implementers choose, particularly where multiple lengths have been tested.

### Limitations and Future Research

Our study relied on interviews and focus groups to explore questions about program modifications asked by FRAME, so we could not systematically quantify how widespread the modifications were (a FRAME reporting area). While some of the modifications, particularly the proactive content adaptations, were applied universally across program delivery, we were unable to determine whether some of the modifications reported by individual implementers were system-wide or unique cases. In some situations, establishing the decision-making process for the modifications was not straightforward, as individuals were not aware of all the steps in the process. Furthermore, asking directly about program modifications did not yield many modifications being reported. This may be due to social desirability bias, as participants expressed that they believed that the program manual should be followed. Additionally, participants may have had difficulty recalling details, as modifications were reported retrospectively. We addressed this by identifying specific areas of modifications in the first round of data collection and focusing on them in the second round.

We could not examine the effects of program modifications on implementation or participant outcomes. Researchers could examine whether certain reactive modifications are associated with differences in outcomes. If sufficient monitoring data from routine practice is collected, this could provide an opportunity to examine naturally occurring variation in individual modifications or clusters of modifications (Bartee et al., 2024; Holtrop et al., 2022). Likewise, future research could examine, such as through multi-arm or factorial trials (Lachman et al., 2019), the effects of shortening and condensing programs proactively as these modifications often occur during scale-up. Comparing outcomes for different program versions and modifications can also help explore which components are key to achieving the intended intervention benefits—an understanding necessary for effective fidelity and scale-up (Caron et al., 2021).

Adding prospective tracking of modifications, such as via surveys or observational coding measures relying on video recordings or live observations, in future studies could provide a more complete picture, particularly if combining multiple methods. However, implementer workload for adding these tasks to their requirements needs to be considered, especially as workload was one of the reasons mentioned for many program modifications found in our study and other studies. There might be a bidirectional relationship between the implementer attitudes to modifying interventions and their perception of job demands, which needs to be disentangled (Zetterlund et al., 2024).

Finally, assessing modification fidelity-consistency is challenging (Stirman et al., 2019). Interventions often do not have a readily available and detailed list of intended functions and forms to compare against to ensure an intervention is implemented as intended. The availability of more documented examples of the processes for assessing fidelity-consistency of intervention modifications would be beneficial for establishing robust and replicable fidelity assessment methods (Caron et al., 2021).

## Conclusion

Drawing on FRAME in designing qualitative data collection and analysis, we have identified a range of context and content modifications and reasons for them, including both modifications we expect to have promoted and challenged intervention fidelity. FRAME provided a helpful framework to conceptualize and document modifications. This study provides a novel glimpse into the modifications made during the scaling up and scaling out of an evidence-based parenting program delivered at a large scale in schools and other community settings in Tanzania.

## Supplementary Information

Below is the link to the electronic supplementary material.

ESM 1 (DOCX 26.9 KB)

## Data Availability

Our data collection tools are available on: https://osf.io/m5fu2/files. The qualitative datasets are not publicly available due to potential for identifying participants.
