# Supplementary material for: Modifications of a Parenting Program in the Context of Scaling-Up and Scaling-Out: Documenting Furaha Teens in Tanzania Using FRAME
Source: Prev Sci. 2025 Oct 30;26(7):1021–32. doi: 10.1007/s11121-025-01852-5 (PMC12627163; doi:10.1007/s11121-025-01852-5)
Supplement: Supplementary file 1 — (DOCX 26.9 KB) [file 11121_2025_1852_MOESM1_ESM.docx]

Table 1s. Intervention modifications documented in the Furaha Teen project

|  | **Modification** | | | **Proactive /**  **reactive** | **Decision-makers** | | **When** | | | **Level of modification** | | | | | **Nature** | | **Fidelity-consistent?** | | | **Reasons for modification** | | |  |
| --- | --- | --- | --- | --- | --- | --- | --- | --- | --- | --- | --- | --- | --- | --- | --- | --- | --- | --- | --- | --- | --- | --- | --- |
| *Context* | | | | | | | | | | | | | | | | | | | | | | |  |
|  | Programme delivered alongside a package of other services | Proactive | | | | Funder, Pact | | Pre-delivery | | | | Project-wide | N/A | | | | | N/A | | | Embedding parenting support within a larger HIV-prevention initiative focusing on adolescent girls |  |  |
|  | Only female adolescents included | Proactive | | | | Funders, Pact | | Pre-delivery | | | | Project-wide |  |  |  |  |  |  |  |  |  |  |  |
|  | Additional classes for male adolescents | Reactive | | | | Facilitator | | During delivery | | | | Individual practitioner |  |  |  |  |  |  |  |  | Feedback from parents questioning exclusive focus on families with girls |  |  |
|  | Including additional family members into the sessions | Reactive | | | | Facilitators | | During delivery | | | | Individual practitioners |  |  |  |  |  |  |  |  | Engagement of participants; acceptability |  |  |
|  | Increased group size | Proactive | | | | CWBSA, Pact Tanzania | | Pre-delivery | | | | Project-wide |  |  |  |  |  |  |  |  | Reach |  |  |
|  | Reduced group size due to COVID | Reactive | | | | Facilitators | | During delivery | | | | Individual practitioners and LIPs |  |  |  |  |  |  |  |  | COVID transmission risk |  |  |
|  | Grouping participants for home visit catch-ups | Reactive | | | | Facilitators | | During delivery | | | | Individual practitioners and LIPs |  |  |  |  |  |  |  |  | Facilitator workload and incentivising group session attendance |  |  |
|  | Personnel: Delivered by school teachers alongside volunteers | Proactive | | | | Facilitators | | Pre-delivery | | | | Project-wide |  | | | | |  |  |  | Increased sustainability, using existing systems |  |  |
| *Content* | | | | | | | | | | | | | | | | | | | | | |  |  |
|  | Added HIV-related content to the intervention manual in consultation with developers | Proactive | | | | Funders, Pact, PLH, CWBSA | | Pre-delivery | | | | Project-wide | Adding elements | | | | | Yes | | | The programme was delivered in a package of services focused on HIV prevention |  |  |
|  | Added condom use demonstration | Reactive | | | | Facilitators | | During delivery | | | | Individual practitioners | Adding elements | | | | | No | | | N/A |  |  |
|  | Translation of the manual into Swahili and adaptation of character names | Proactive | | | | CWBSA, Pact | | Pre-delivery | | | | Project-wide | Tailoring | | | | | Yes | | | Cultural contextualisation for acceptability among families in Tanzania |  |  |
|  | Verbal interpreting into additional languages, adding local songs | Reactive | | | | Facilitators | | During delivery | | | | Individual practitioners | Tailoring | | | | | Yes | | | Further cultural contextualisation for improved acceptability |  |  |
|  | Frequency of sessions (two or three times a week instead of one) | Reactive | | | | LIPs, facilitators, families | | During delivery | | | | LIPs, individual practitioners | Condensing | | | | | No | | | Funding timelines; COVID delays |  |  |
|  | Longer sessions including re-caps of past materials | | Reactive | | | LIPs, facilitators | | | During delivery | | LIPs, individual practitioners | | | Repeating | | Yes | | | COVID related pauses in delivery, modification to help with re-engagement of participants | | | | |
|  | Number of group sessions (merging sessions together) | | Reactive | | | Pact Tanzania, LIPs, facilitators | | | During delivery | | LIPs, individual practitioners | | | Condensing | | No | | | Funding timelines; COVID delays | | | | |

**Coding details**

“Nature” and “fidelity-consistent” assessment of modification apply to content modifications only. “Nature” is categorised using categories specified in FRAME for content modification.

When: Pre-delivery, during delivery

Decision-makers: Families, facilitators, LIPs (implementing organisations), Pact Tanzania (coordinating organisation), CWBSA (Clowns Without Borders South Africa, training organisation), funder

Level of modification options: individual practitioners, LIPs (implementing organisations), project-wide
